# Developmental transcriptome profiling uncovered carbon signaling genes associated with almond fruit drop

**DOI:** 10.1038/s41598-020-69395-z

**Published:** 2021-02-09

**Authors:** Chunmiao Guo, Yu Wei, Bo Yang, Mubarek Ayup, Ning Li, Jun Liu, Kang Liao, Huan Wang

**Affiliations:** 1 College of Forestry and Horticulture, Xinjiang Agricultural University, Urumqi, 830052 China; 2 Biotechnology Research Institute, Chinese Academy of Agricultural Sciences, Beijing, 100081 China; 3 National Key Facility for Crop Resources and Genetic Improvement, Institute of Crop Science, Chinese Academy of Agricultural Sciences, Beijing, 100081 China; 4 Institute of Horticultural Crops, Xinjiang Academy of Agricultural Sciences, Urumqi, 830091 China

**Keywords:** Transcriptomics, Gene expression profiling

## Abstract

Almond is one of the most featured nut crops owing to its high nutritional value. However, due to three different waves of flower and fruitlet drop, fruit drop is a major concern for growers. In this study, we carried out a time-course transcriptome analysis to investigate gene expression differences between normal and abnormal fruitlet development. By de novo assembly analysis, we identified 33,577 unigenes and provided their functional annotations. In total, we identified 7,469 differentially expressed genes and observed the most apparent difference between normal and abnormal fruits at 12 and 17 days after flowering. Their biological functions were enriched in carbon metabolism, carbon fixation in photosynthetic organisms and plant hormone signal transduction. RT-qPCR validated the expression pattern of 14 representative genes, including *glycosyltransferase like family 2*, *MYB39*, *IAA13*, *gibberellin-regulated protein 11-like* and *POD44*, which confirmed the reliability of our transcriptome data. This study provides an insight into the association between abnormal fruit development and carbohydrate signaling from the early developmental stages and could be served as useful information for understanding the regulatory mechanisms related to almond fruit drop.

## Introduction

Almond (*Prunus dulcis* (Mill.) D.A. Webb. syn. *Prunus amygdalus* Batsch), is the most important temperate nut crop regards to commercial production, and several hypotheses indicated that it originated from Iran and specifically adapted to harsh climates^1,2^. Despite its wide adaption, almond is usually cultivated in areas with Mediterranean climate. The important commercial production regions include Asia, Mediterranean countries, American and Australia. Main growing regions in Asia are Iran and Turkey^2^. In China, almond is mainly cultivated in western China in Xinjiang province and becomes one of the most important economic nut crops in the area^2,3^. The almond production of China in 2017 was 51,953 tons^4^. In almond, fruit drop occurs at three distinct stages with different reasons^2^. The first drop takes place just after bloom or even in the late stages of bloom on malformed flowers, mainly caused by deficient pistil development. The second drop occurs after bloom mainly on unfertilized flowers. The flowers may become pollinated but not fertilized because the pollination stimulates the initial development until they are pea sized. Some fertilized flowers may also drop due to cytological defects. The third drop is physiological and often referred to as ‘June drop’, which is probably caused by nutrimental competition, and at this stage the fruits going to fall normally having a smaller size and yellowish epiderm^2^. In production, a number of flowers/fruit drop is a need for almond production since not all flowers could develop into ripe fruits^2,5^, however, fruit drop also takes place in pollinated fruits and results in very low fruit sets, whatever the blooming time and other conditions during the growing stage^5^. In other fruits, it is also a factor that affects fruit development^6^. The abscission is a developmental process that mediated by both internal and environmental cues^7^. Endogenous phytohormones are demonstrated to contribute to fruit abscission, among which auxin is an important regulatory factor^8^. In the meantime, the external nutrient supply is suggested to take a major regulatory role. A few studies have revealed that the nutrient competition between fruitlets and leaves result in fruit abscission^9,10^. Much efforts have been done on peach, apple, longan and other fruit trees, and it is implicated that the sucrose accumulation and its metabolism are the main factors that cause abnormal fruitlets^11–14^.

Moreover, carbon balance may be negatively related to shading, and the combined effects of photosynthetic inhibitors and shading caused apple fruit abscission^15–17^. Nicolás and collaborators suggested that C-transfer could happen between new branches and fruits^18^. Nzima and collaborators further proved that the developing fruits have a drop upon carbohydrates competition with other organs of the tree^19^. What’s more, the increment of enzymes functioning in sugar inversion was related to orange fruit abscission during fruitlet development^13^. In litchi, high ethylene production and low glucose levels were proposed to contribute to the increasing young fruit abscission rate^20^. Despite of these facts, the molecular understanding of the regulatory genes of carbohydrate metabolism involved in almond fruitlet abnormal development and physiological drop is still elusive.

High throughput sequencing technologies have revolutionized the genomic and transcriptomic studies. In particular, improved algorithms are now available for de novo reassembling the transcriptome of a non-model plant species without a valid reference genome sequence^21,22^. Subsequently, the assembled transcripts can be used to profile gene expression patterns, reconstruction of co-expression networks, and characterize genetic variations in regions. Currently, RNA sequencing (RNA-seq) has been widely exploited in almond to uncover the molecular bases in response to cold, drought and other stresses^23–25^, as well as in the understanding of its self-incompatibility, flower bud dormancy and domestication history^26–28^. However, the transcriptome analysis associated to almond abscission has not been reported yet.

The aims of this study were to profile the gene expression levels in normal and abnormal fruitlets and to identify differentially expressed genes (DEGs) at different developmental stages, and to understand the gene regulatory networks underlying fruit abscission in almond. To this aim, a genome-wide time-course transcriptome analysis of almond using RNA-seq and validated by RT-qPCR analysis on representative genes was carried out.

## Results

### Phenotype evaluation

**Figure 1:**
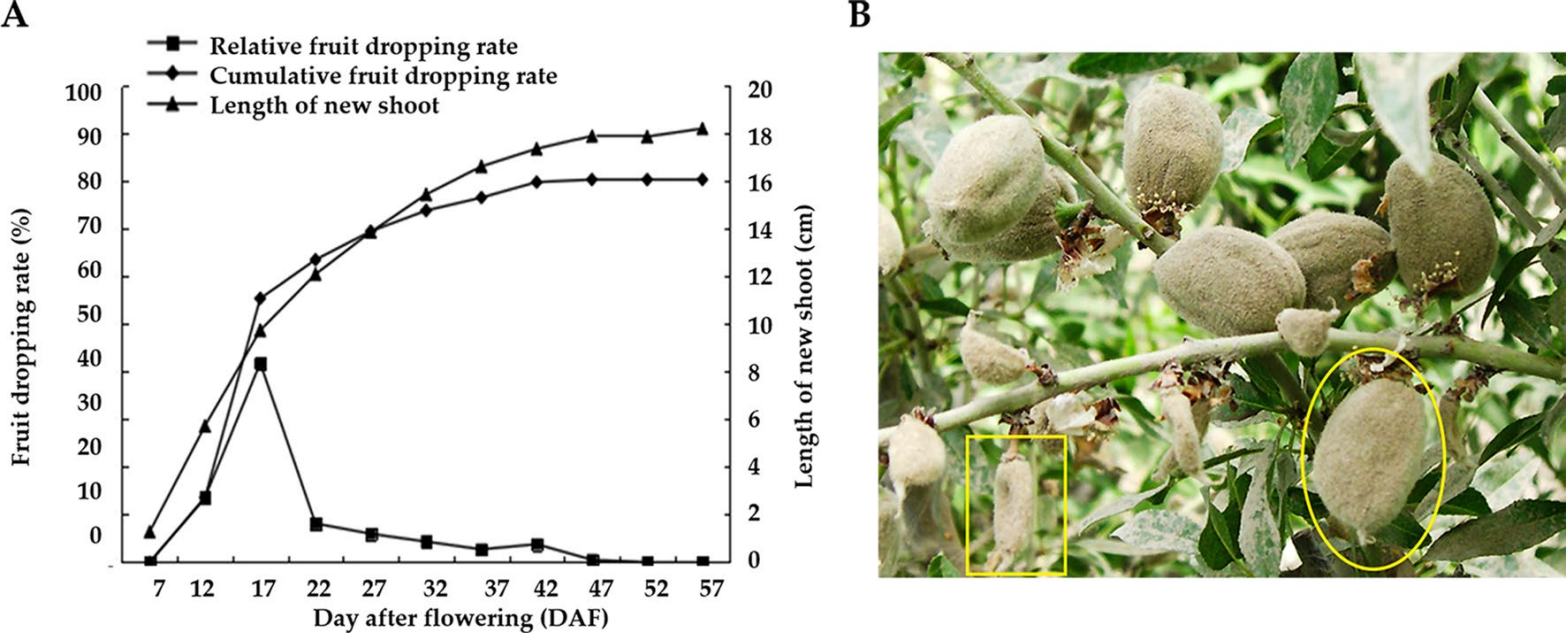
(**A**) Dynamic changes of almond shoots increment and fruit dropping rate; (**B**) Growth state of the fruits at 32 DAF. The yellow circle locates the normal fruits, and the yellow box locates the abnormal fruits.

Our field study shows that the full-bloom stage of almond starts at the end of March in Xinjiang and the shoot buds also sprout at this stage. The fruit setting normally occurs at the 7th day after flowering (DAF). We investigated the new shoot elongation and the fruitlet dropping rate that at the period from 7 to 57 DAF. As shown in Fig. 1A, from 7 to 37 DAF, the new shoots elongated most, and the fruitlet dropping rate increased significantly. Particularly, from 7 to 22 DAF (5th April to 20th April), the average length of the new shoot was increased from 1.28 cm to 12.11 cm (Fig. 1A), which comprised 68.03% of the whole shoot length. During this stage, the fruitlets enlarged remarkably, and the dropping rate increased dramatically (Figure S1). After that, from 22 to 57 DAF (20th April to 25th May), the new shoots turned to grow slowly, meanwhile, the fruitlets dropping rate increased slowly. Moreover, we conducted the morphological evaluation of normal and abnormal almond fruitlets at the period from 12 to 42 DAF (Figure S2). The fruit weight, the longitudinal and transversal diameters of abnormal fruits were all significantly less than that of normal fruits when comparing parallelly. Consistent with the transition time the shoots turned to grow slowly, from 22 DAF the normal fruits enlarge significantly (Figure S2).

Given that the period from 12 to 37 DAF was the main stage that accounted for the fruitlet dropping and the new shoot elongation, we collected samples at six stages, including 12, 17, 22, 27, 32, 37 DAF. At each stage, we sampled normally developed and diapause atrophy fruits (abnormally developed) in the same branch. The abnormally developed fruits were the ones that most likely to shed in the following days. The characteristics of normal and abnormal fruitlets at 32 DAF are shown in Fig. 1B.

### De novo assembly and annotation of almond unigenes

**Figure 2:**
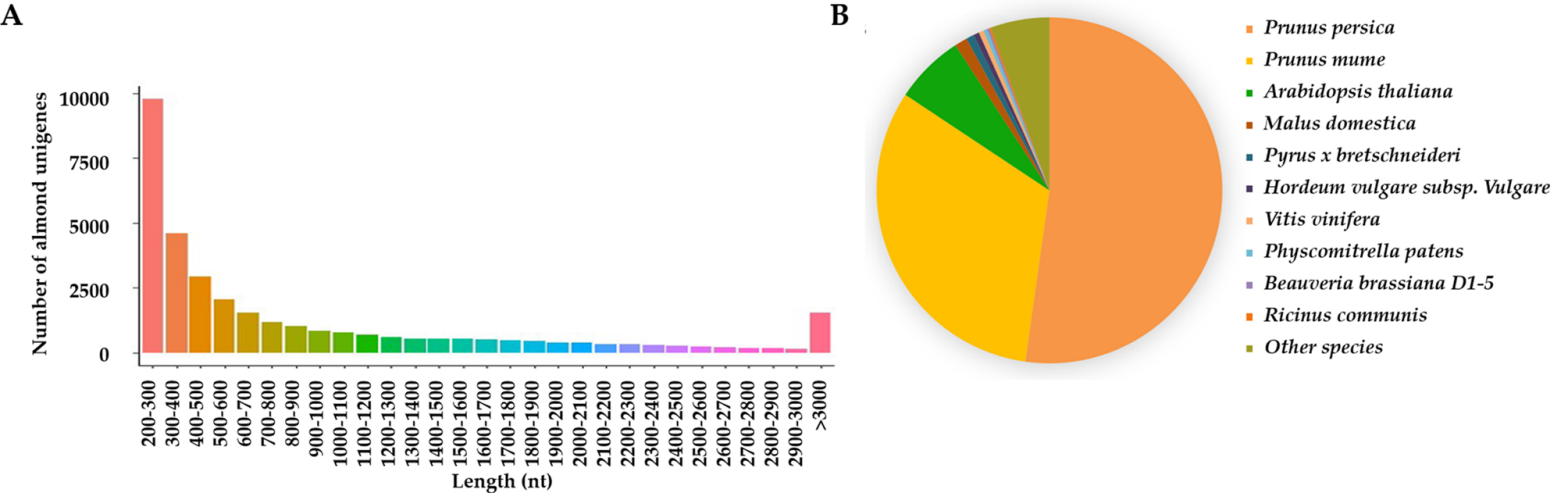
(**A**) Length distribution of almond representative unigenes; (**B**) Pie chart of almond unigene homologs.

To investigate the regulatory genes that correlated to almond fruit development at transcriptome level, we sequenced normal and abnormal fruits at 12, 17, 22, 27, 32, 37 DAF, respectively. RNA samples with three biological replicates were prepared and sequenced using Illumina sequencer with paired-end protocol. After filtering out the low-quality reads (see “Methods”), Q30 and GC content of the clean data were calculated as shown in Table S1. In total, we obtained 296,587,721 clean reads. Following, we performed de novo transcriptome assembly using Trinity^29^ and obtained 70,500 contigs, from which 33,577 representative unigenes with an N50 of 1,698 nt were generated. The length of unigenes ranged from 201 to 28,660 nt with the average length 1,026 nt (Fig. 2A). These results confirmed that the throughput and data quality were good and qualified for following analyses.

We scanned putative peptide sequences based on the nucleotide composition using TransDecoder (https://transdecoder.sourceforge.net/). In total, 26,996 unigenes could encode ORFs with at least 100 amino acids. Using BLASTP and BLASTX, we annotated the unigenes by aligning the assembled transcripts and predicted peptide sequences to NCBI NR and Swiss-Prot databases. In total, we identified 22,182 unigenes containing significant matches to the annotated genes/proteins in at least one database. Additionally, we also aligned the unigenes to Pfam, COG, eggNOG, GO and KEGG databases (Table S2). The detailed results were shown in Supplemental Table S3, which could be served as a reference annotation database for the future up studies. We summarized the best-hit species from BLASTX results and found that the top two matched species are *Prunus persica* (52.24%) and *Prunus mume* (32.09%) (Fig. 2B). Genetic and evolutionary studies of peach indicated that peach and almond share common characteristics in precocity, organization and perenniality^30–32^, indicating the accuracy of our annotation analysis in almond.

### Profiling gene expression during fruit development

**Figure 3:**
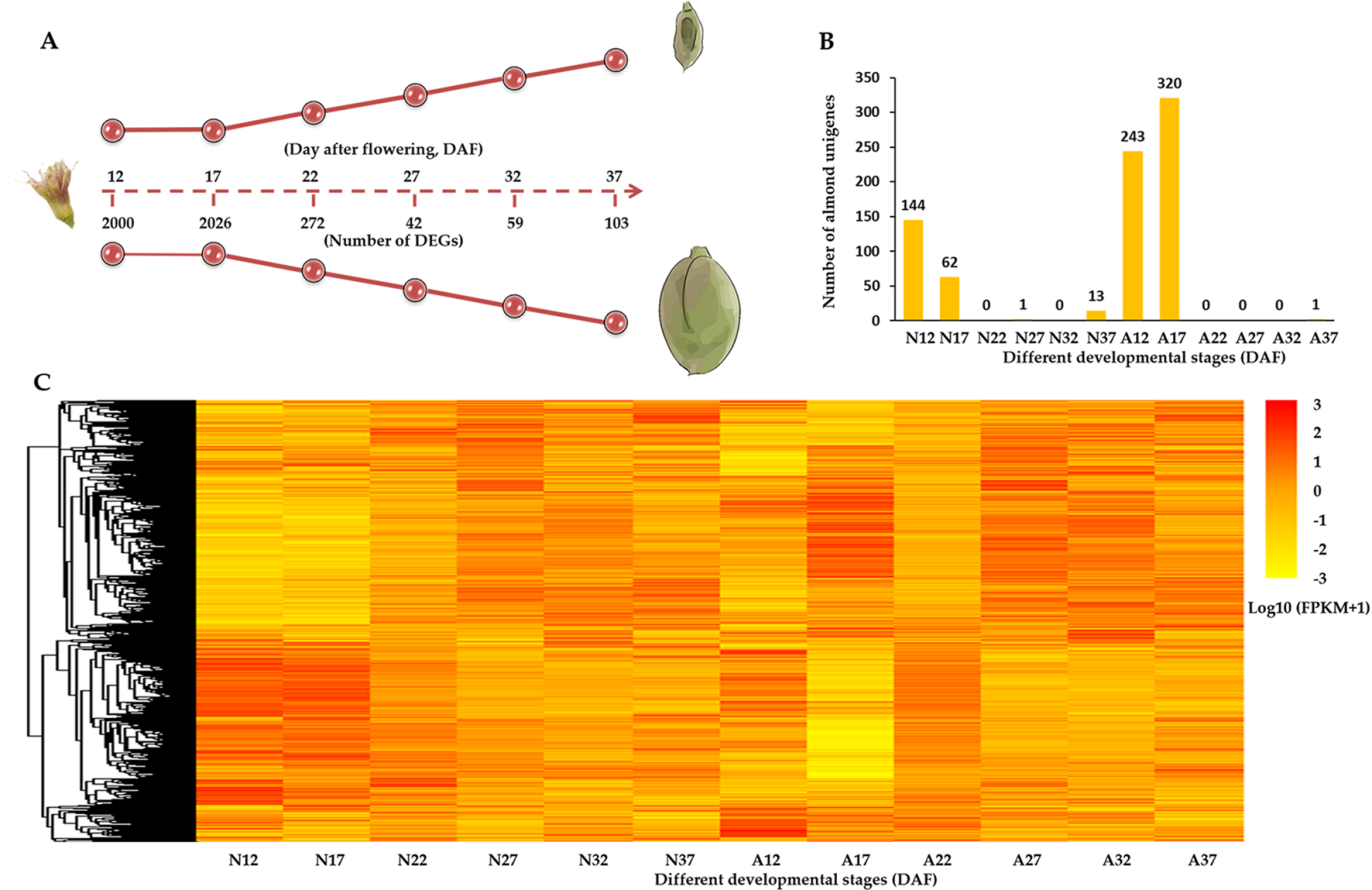
(**A**) The schematic graph of this study (This figure was drawn by Xinchang Li); (**B**) The number of developmental stage specific genes; (**C**) The heatmap of total DEGs. ‘N12′ represents 12 DAF of normal fruits; ‘A12′ represents 12 DAF of abnormal fruits.

Next, we measured the expression levels of unigenes by calculating Fragments Per Kilobase of transcript per Million fragments mapped (FPKM)^33^. Using DESeq2^34^, we normalized FPKM values and searched for DEGs between normal and abnormal fruits at specific time points as well as between different time points in normal or abnormal samples (log2 value of fold change ≥ 1 and *P*-value < 0.05) and identified 7,469 DEGs in total (Table S4). Of them, 2,084 DEGs were found between normal and abnormal fruits at each time point (Fig. 3A). Note that 220 and 564 genes were exclusively detected in the normal and abnormal almond fruits, respectively (Fig. 3B). And normal–abnormal differentially expressed genes were specifically enriched at 12 and 17 DAF.

Moreover, there were 5,843 and 6,245 developmental stage specific genes in normal and abnormal fruits, respectively (Figure S3). Comparing each two time points, the most apparent transcriptome change was also observed at 12 and 17 DAF, especially that in abnormal samples (Fig. 3C). This molecular evidence was in line with the phenotype observation that the increment of both fruitlet dropping rate and shoot elongation retarded from 17 DAF, especially the fruit dropping rate reached the max value at this stage. Together, these evidences suggest a crucial transition in early stage of developmental process, which in turn proves the importance of the regulatory genes at 12 and 17 DAF stages in the fruit development.

**Figure 4:**
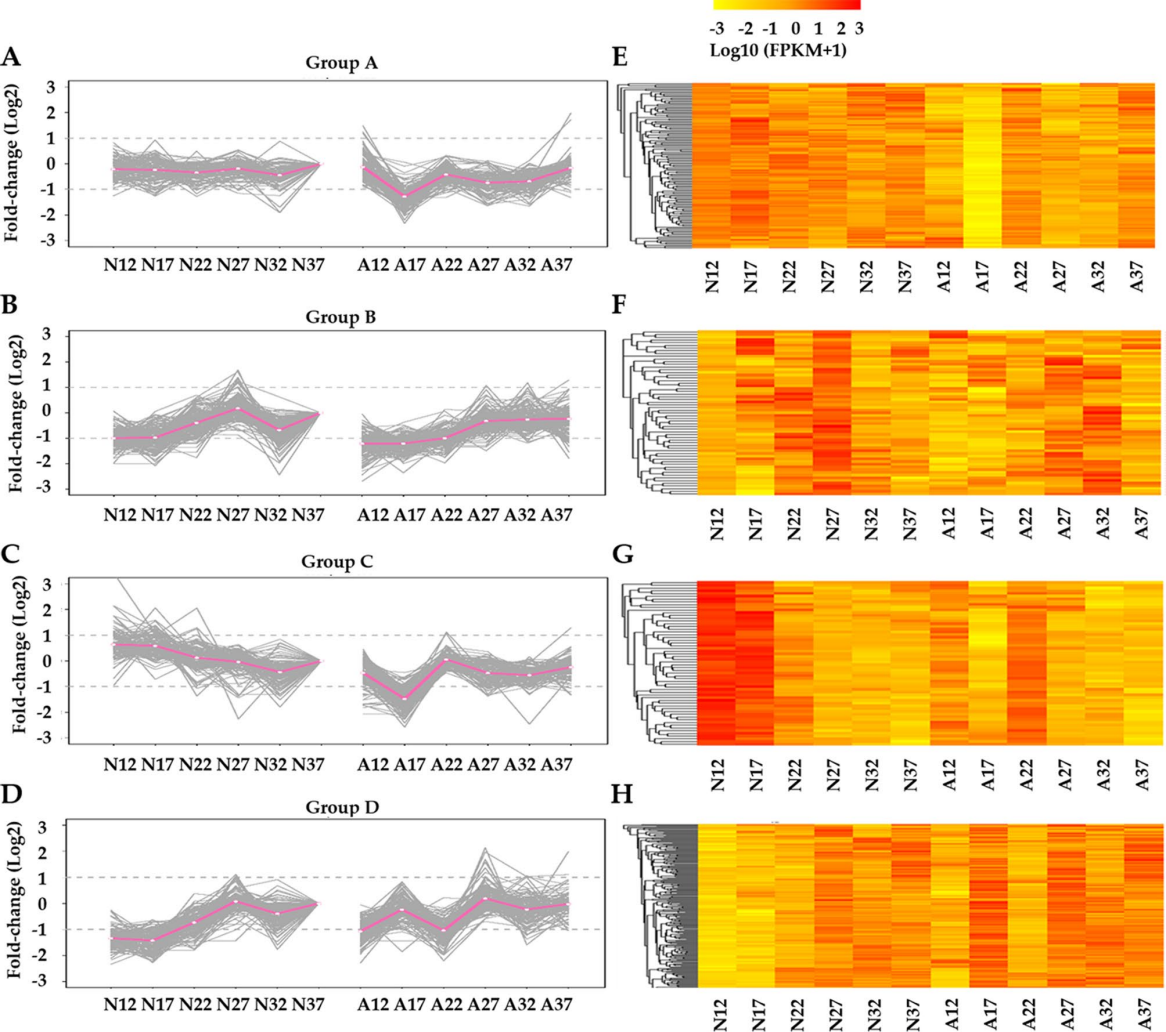
The expression patterns and heatmaps of representative DEGs groups. (**A**–**D**) y-axis gives log2 value of fold change. (**E**–**H**) heatmap presents log10 value of FPKM. The expression patterns were performed using 12 DAF as the baseline. “N12” represents 12 DAF of normal fruits; “A12” represents 12 DAF of abnormal fruits.

To compare gene expression patterns between normal and abnormal samples, all DEGs were assigned for *K*-means Clustering analysis based on the similarities in their expression profiles (*P*-value < 0.05). And 7,469 DEGs were classified into 120 clusters (Table S4). Comparing expression profiles in normal and abnormal samples, 536 DEGs presented similar expression profiles between normal and abnormal fruits (Figure S4A). And 6,070 DEGs showed different expression patterns between normal and abnormal samples. Among them, 2,263 genes exclusively presented developmental specific expression patterns in normal or abnormal fruits (Fig. 4A,E), and others presented different expression patterns between normal and abnormal samples (Fig. 4B–D,F–H and Figure S3B).

Four representative clusters were presented in Fig. 4. In representative group A, genes showed relatively stable expression level from 12 to 37 DAF in normal fruits, while in abnormal fruits these genes displayed a sharp down-regulation at 17 DAF and slightly increased afterwards. This group contained a number of genes that previously reported to function in fruit development and ripening^35–37^, including the genes encoding auxin associated proteins (auxin canalisation and auxin response factor 9) and transcription factors (GUF1 homolog, SPATULA, zinc finger protein ZAT9, TCP2 and Myb/SANT-like DNA-binding domain), as well as probable protein phosphatase 2C. In group B, the genes expression was firstly up-regulated and peaked at 27 DAF and then down-regulated in the normal fruits, while in the abnormal fruit these gene slightly increased from 12 to 32 DAF and decreased afterwards. These genes including the ones encoding auxin response factor 6, basic leucine zipper 61 and glycosyl hydrolase family 10. In group C, the expression of genes gradually decreased in normal fruits underlying the developmental stages, while they fell sharply at 17 DAF and went up at 22 DAF then gradually decreased in abnormal fruits. These genes including three UDP-glucosyl transferases, one Alpha-amylase 3 encoding genes, as well as several secondary metabolites biosynthesis associated genes. In group D, genes expression in normal fruits first peaked at 27 DAF and then fell at 32 DAF and later raised again, while in the abnormal fruit these genes presented three peaks at 17, 27 and 37 DAF, respectively. These genes including several transcription factors (TFs) (e.g., *WRKY49*, *MYB44*, *Trihelix TF GT-4*, *PCL1*, *ethylene-responsive TF RAP2-12* and *bZIP43*). The abundance of these representative genes was also shown in the heatmaps displayed in Fig. 4. Consistent with the enrichment of stage-specific genes at 17 DAF (Fig. 3) as well as the stage transition at 17 DAF observed in phenotype (Fig. 1A), the clustering analysis also illustrates a clear stage-specific difference at 17 DAF between normal and abnormal fruit samples.

### Functional enrichment of differentially expressed genes

**Figure 5:**
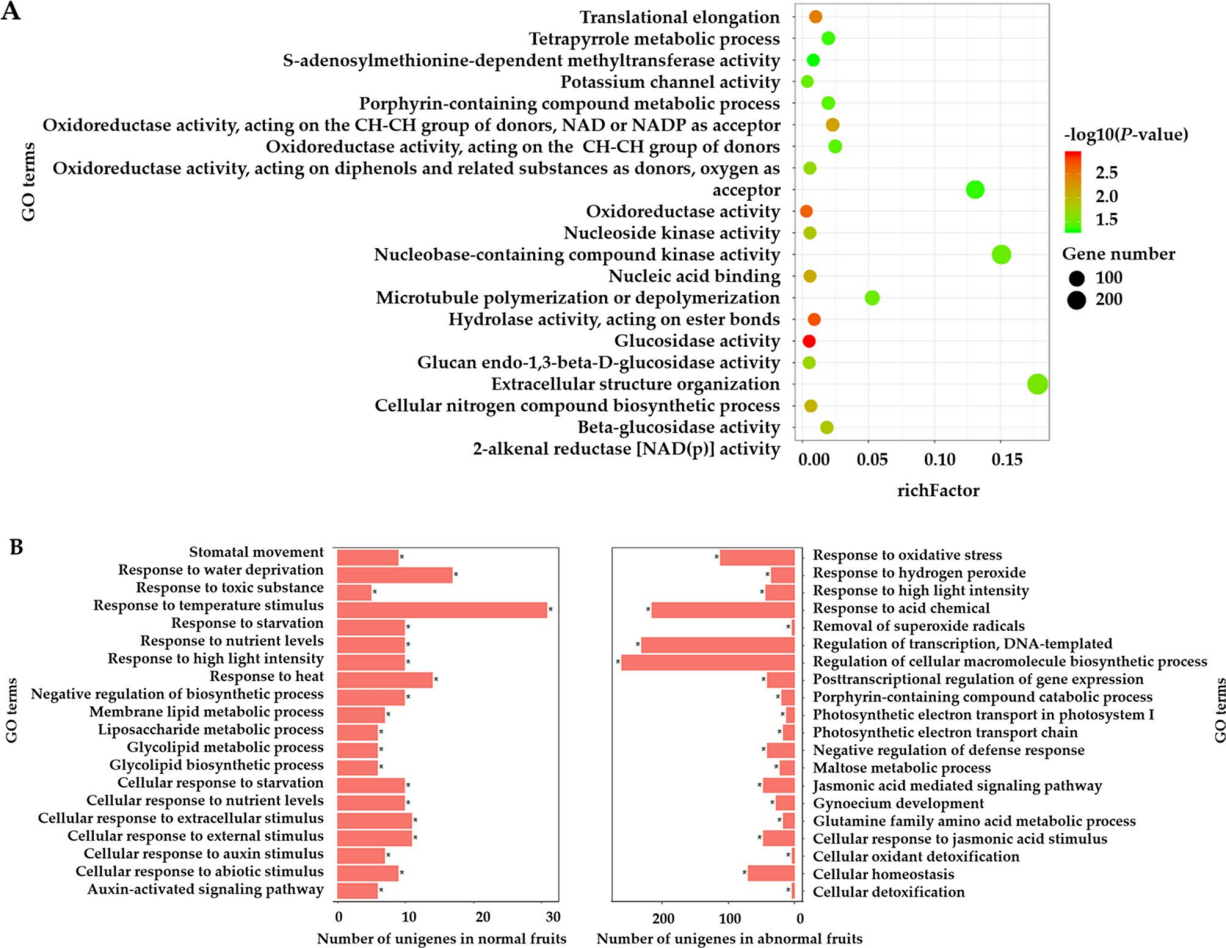
(**A**) Functional enrichment analysis of normal-abnormal differentially expressed genes at 17 DAF; (**B**) Functional enrichment analysis of stage-specific genes in normal and abnormal fruits, respectively.

Differentially expressed genes might perform biological roles related to fruit drop. Therefore, we performed Gene Ontology (GO) enrichment analysis to investigate the functions of the differentially induced/repressed genes using agriGO, GOEAST and ggplot2^38–40^. Given the amount of stage-specific DEGs expressed at 17 DAF, we performed GO enrichment analysis of normal-abnormal differentially regulated genes at 17 DAF. As presented in Fig. 5A, we detected significantly enriched terms, including "glucosidase activity”, “oxidoreductase activity” and “beta-glucosidase activity”, at 17 DAF. Together, these results suggest that diverse regulatory mechanisms including multiple carbohydrate and plant hormones signaling pathways dominate in the fruits development. For developmental stage specific genes, a total of 1,859 and 2,225 DEGs from normal and abnormal fruits were assigned to GO terms and were enriched into 20 and 39 GO terms, respectively (hyper geometric test, *P*-value < 0.05). The top 20 enriched GO terms of normal and abnormal samples are shown in Fig. 5B. Developmental stage specific DEGs of normal fruits were significantly enriched in response to nutrient levels and auxin stimulus, whereas the developmental stage specific DEGs of abnormal samples were mainly enriched in response to oxidative stress, jasmonic acid mediated signaling pathway and maltose metabolic process. Alternatively, varied GO terms were observed between normal and abnormal samples.

**Figure 6:**
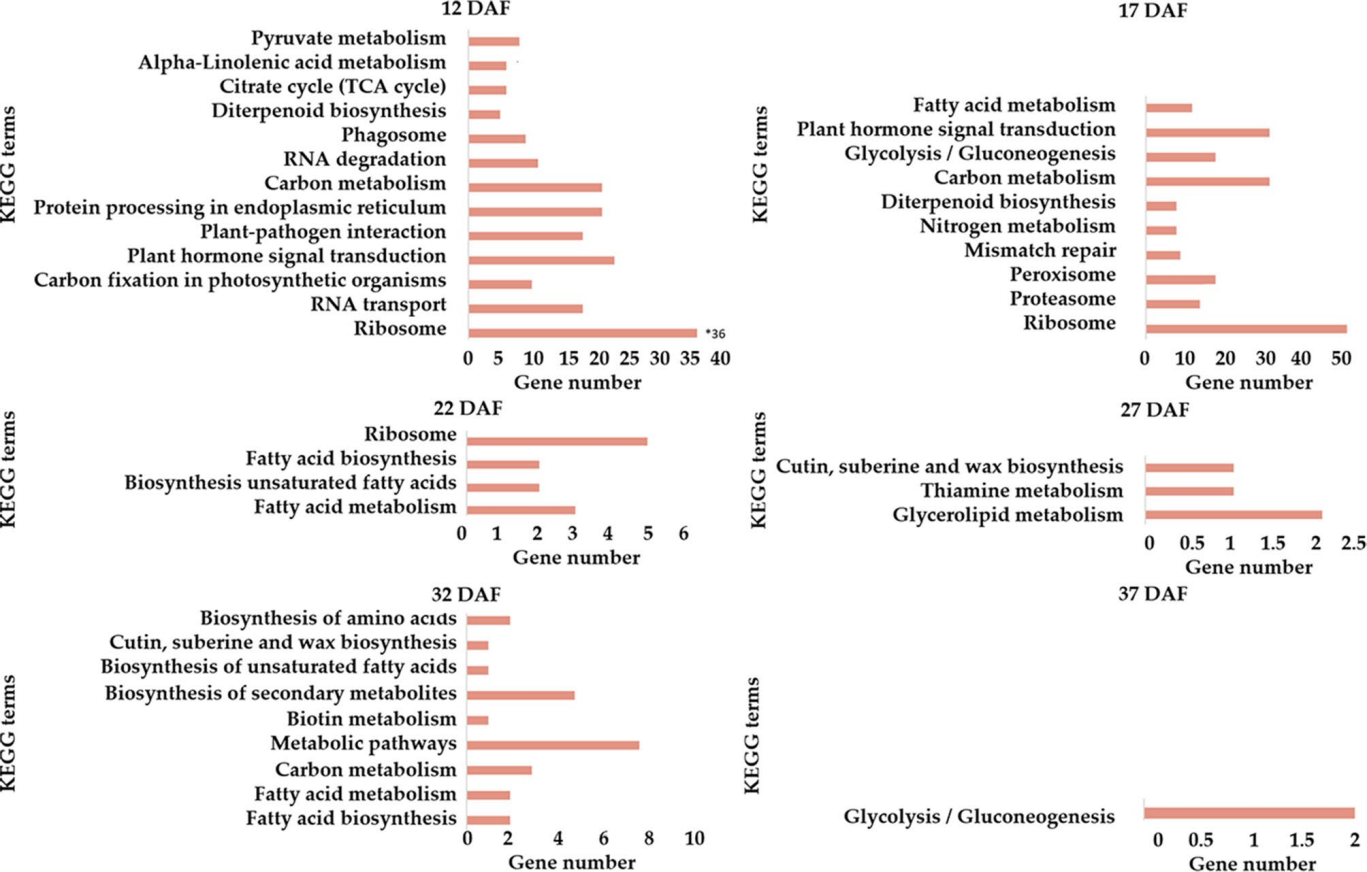
KEGG enrichment analyses of DEGs between normal and abnormal almond at six stages.

To further dissect DEGs-involved pathways, normal-abnormal DEGs at different stages were subjected to KEGG enrichment analysis with KOBAS 3.0 . There were 192 and 203 unigenes at 12 and 17 DAF assigned to 13 and 10 KEGG pathways in the KEGG database, respectively, and 43 genes fell into 17 KEGG pathways at other periods (Fig. 6). Interestingly, we found the highly enriched pathways at 12 DAF were related to “protein processing in endoplasmic reticulum”, “RNA transport” and “plant hormone signal transduction” (including IAA13*,* DN26918_c0_g1*).* While the majority of genes at 17 DAF was classified into pathways for “carbon metabolism” (including *fructose-1–6-bisphosphatase, DN18563_c0_g1; lactate/malate dehydrogenase*, *DN15548_c0_g1,* Figure S5), “glycolysis / gluconeogenesis” (including *aldose 1-epimerase*, *DN13867_c0_g1)* and “plant hormone signal transduction” (including *auxin-induced protein 15A, DN18673_c0_g1*; *auxin-responsive protein IAA13*, *DN26918_c0_g2).* The pathway for “peroxisome” (including *peroxidase44*, *DN24026_c1_g1*) was also enriched at 17 DAF. In addition, several carbohydrates associated DEGs (e.g., *malate dehydrogenase, DN15548_c0_g2, succinate dehydrogenase, DN18450_c0_g1*, *citrate synthase 4, DN23508_c0_g1 and NADP-dependent malic enzyme 2, DN7242_c0_g1)* were annotated to be involved in energy production and conversion at both 12 and 17 DAF. We also observed that carbohydrate-related pathways or metabolisms were enriched across all the six stages, including the fatty acid metabolism at 22 DAF, glycerolipid metabolism at 27 DAF, carbon metabolism at 32 DAF, as well as the term of glycolysis/gluconeogenesis at 37 DAF, confirming that the carbohydrate-related pathway or metabolism might respond to fruitlet development in almond, which is in line with the findings in litchi and other fruits^13,19,20^. Differently, the pathway of “cutin, suberine and wax biosynthesis” was classified at both 27 and 32 DAF. As we know, cutin and suberine are polymer matrices for cell wall barriers that function in protecting plants from stresses and controlling plant morphology^41^.

### Experimental validation of selected DEGs by RT-qPCR at different periods

**Figure 7:**
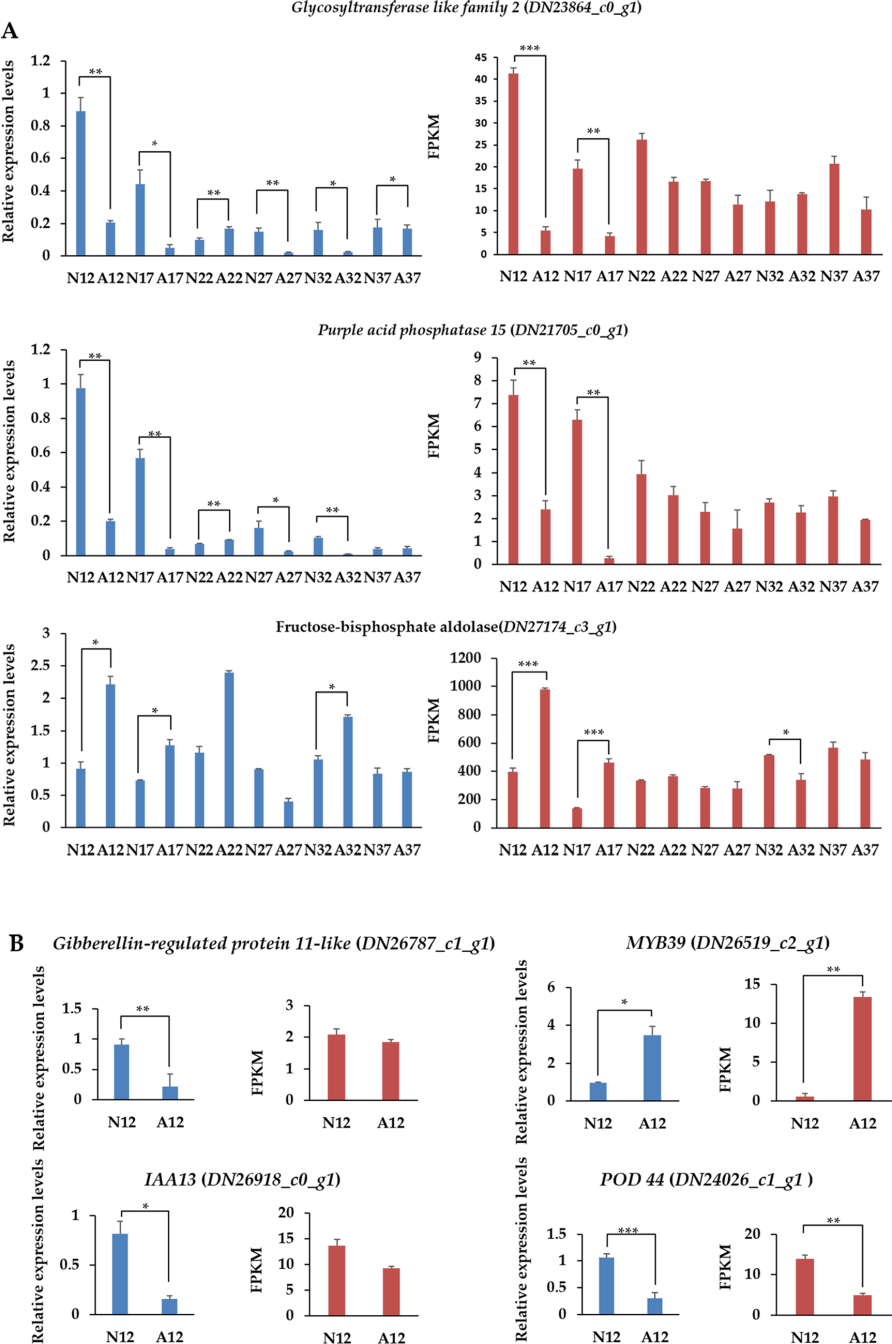
Relative expression analysis of six randomly selected DEGs related to almond fruitlet abnormally development. (**A**) Overall expression levels of *glycosyltransferase like family 2* and *purple acid phosphatase 15* across different developmental stages; (**B**) Expression levels of DEGs at specific stages. Bars give standard errors (n = 3). *N* normal, *A* abnormal. Blue, RT-qPCR analysis; red, RNA-seq analysis. *P*-values indicate statistical significance (*P < 0.05, **P < 0.01, ***P < 0.001).

In order to validate the transcriptional expression levels of differentially expressed unigenes in normal and abnormal fruits determined by RNA-seq data, we employed quantitative real-time PCR (RT-qPCR) assay to assess 14 randomly selected DEGs (Fig. 7 and Figure S6A). The RT-qPCR results showed overall consistent profiles with the RNA-seq data (the overall correlation coefficient of *R* = 0.754, *P*-value < 0.001, Figure S6B). These results demonstrate that there is a good consistence between RNA-seq data and RT-qPCR results in our study, which confirmed the reliability of our RNA-seq data analysis.

Glycosyltransferases (GTs) are enzymes that creating a glycosidic bond between a sugar molecule and a specific acceptor, which can be other sugars, proteins, lipids and small molecules^42^, and plant cell walls are composed of complex polysaccharides that require many GTs for their synthesis^43^. Comparing to normal fruits, both *glycosyltransferase like family 2* (*DN23864_c0_g1*) and *PAP15* (*DN21705_c0_g1*) were down-regulated in abnormal fruits at 12 and 17 DAF. In addition, there was a sharp reduction at 17 DAF and following a re-increment at 22 DAF during the whole abnormal development, and the expression profiles of both genes in abnormal samples were mostly lower than those in normal fruits. Purple acid phosphatase (PAP) is an enzyme family that demonstrates a violet color in solution, it confers shoot growth stimulation, enhances osmotic stress tolerance and ABA insensitivity. PAPs also present peroxidase activity and function in the production of ROS in plant response to stresses^44^. They may act as multifunctional enzymes and play crucial roles in plant growth and development under normal or stressed environment^45^. *Arabidopsis PAP15* exhibits phytase activity and could function in enhancing cell wall synthesis by supplying additional myoinositol from phytate^45^. The overall reduction of *glycosyltransferase like family 2* and *PAP15* at six stages illustrates that the fruit development including the cell wall modifying related processes were influenced after the initiation of the abnormal development signaling in early stages.

In addition to the decreased expression level of genes encoding enzymes, the activity of a transcription factor *MYB39* (*DN26519_c2_g1*) was highly increased in abnormal fruit at 12 DAF. Multiple members from MYB and bZIP families have been previously characterized to be critical regulators to developmental or environmental stimuli in apple^46^, strawberry^47^ and peach^48,49^, and *MYB39* was indicated to regulate the flavonoid biosynthetic pathway in strawberry^50^. Here, the identification of this gene in almond indicates its possible regulatory role in regulating the almond fruit development via a flavonoid associated pathway.

## Discussion

In this study, we constructed a transcriptome of normal and abnormal fruitlets from 12 DAF till 37 DAF. A total of 33,577 unigenes were assembled with a N50 of 1,698 nt. Since there is no reference genome of almond, we further annotated the almond unigenes to public databases (COG, GO, KEGG, KOG, Pfam, Swiss-Prot, eggnog and NR) with Trinotate pipeline. There were 22,182 (66.1%) unigenes annotated, indicating that one third of the assembled unigenes own no significant known homologs in close species, as suggested in Plum^51^. Among this information, the annotation using NR database showed that the most matched species is *Prunus persica* (52.24%), which is a genetically and evolutionarily correlated species with almond. Hence, the unannotated unigenes could be almond specific with unknown functions, and the corresponding transcriptome information of almond could be served as useful datasets for almond further study in fruit developmental processes.

Fruit is an organ that depends mainly on nutrients supplied by other parts of the plant. In the early stage of fruit development, the growing shoots and fruits compete for limited nutrient and carbohydrate resources^9^. Carbohydrates like sugars play crucial roles in plant development by fueling cellular carbon and energy metabolism, as well as by being signaling molecules^52^. The assimilate movement of carbohydrates was tracked using ^14^C, which proved a competition of carbohydrates between the process of shoot elongation and fruit development after anthesis^10^. Artificial shading could lead to fruit abscission owing to the decreasing photosynthesis, which further aggravates the nutrient competition among organs^11,53^. Considering the importance of optimal fruit set^54,55^, relatively low fruit set ratio and high abscission percentage in almond call for more researches to address the related developmental mechanisms^5^. Many biological studies have been conducted underlying physiological and molecular mechanisms associated with fruit abscission on apple^11^, litchi^12^, Macadamia^56^ and other fruits, however, there is no molecular understanding of the abnormally developed fruitlets signaling at the transcriptional level in almond. Therefore, the main objective of our study was to identify genes that participate in almond fruit developmental process that are expected to be differentially expressed following anthesis.

In this study, we found that a number of differentially expressed unigenes with known function fell into the categories associated with carbon metabolism, carbon fixation in photosynthetic organisms and plant hormone signal transduction. Furthermore, the carbohydrate-related pathways or metabolisms were found enriched across all the six stages. Consistently, in litchi, the GO and KEGG analyses revealed that diverse metabolisms and pathways including carbohydrate metabolism, phytohormone signals, transcription factor activity and cell wall modification were significantly enriched among the genes involved in the early fruit abscission process^20^. Hence, we propose that the carbohydrate-related pathways or metabolisms might also take dominant roles in the abnormal fruitlet development in almond. In the meantime, we observed DEGs involved in carbon fixation in photosynthetic organisms and plant hormone signal transduction were enriched at 12 and 17 DAF, while the number of these genes decreased in later stages. These results suggest that the energy consumption of carbohydrates for maintaining the fruit development occurred in the early stage, and the signal already initiated at 12 DAF. This observation is consistent with the fruit abscission study on litchi, where younger fruitlets were used and the energy consumption occurred at a relative late stage^20^.

Cutting off carbohydrate supply by physical treatments induced 100% longan fruit abscission, and a burst of reactive oxygen species (ROS) occurred in the plasma membrane and cell walls following treatments. In the meantime, activities of antioxidant enzymes including superoxide dismutase, catalase, and POD were all enhanced^14^. In this study, the expression of *POD44* (*DN24026_c1_g1*) in abnormally developed samples was increased comparing with that in normal samples at 17 DAF. There was a peroxisome KEGG pathway enriched at 17 DAF, moreover, the clustering analysis and the phenotype stage transition present a major transition occurring at 17 DAF in the abnormally developed fruit.

Additionally, we identified a few auxin, ethylene and ABA responsive genes, as well as several gibberellins regulated genes. Compared with the expression in normal fruit at 12 DAF, the gene expression of both *IAA13* (*DN26918_C0_g1*) and *gibberellin-regulated protein 11-like* (*DN26787_C1_g1*) in the abnormal sample was strongly declined. However, at 12 DAF, the abscisic acid receptors *PYL4* (DN23029_c0_g1), *PYL8* (*DN22391_c0_g1*), *PYR1* (DN24477_C0_g1) showed relatively higher expression levels in abnormal fruits than in normal fruits. Plant hormones have long been well studied for their importance in fruit development, and this involves auxin, gibberellin, cytokinin, abscisic acid (ABA) and ethylene that undergo major change^57,58^. In general, ethylene and jasmonic acid accelerate fruit abscission, while auxin and gibberellin inhibit abscission^59^. It is widely believed that the endogenous balance of auxin and ethylene plays an important role in fruit abscission. In litchi, the auxin responsive genes were repressed in the dropped fruit induced by girdling plus defoliation^20^. In addition, a previous study on the relationship between the “June drop” of almond fruits and the fruit IAA levels proposed that the decreased IAA levels lead to a higher percentage of fruit abscission^60^. Therefore, apart from the dominant role of carbohydrates in fruit development, the plant hormones of IAA and GA are also likely to participate in the regulation of abnormal development.

Based on these results, we speculate that the developmental signals in almond fruitlet initiates at an earlier phase. Fruits may first perceive carbohydrate stress caused by the competition between fruit enlargement and rapid shoot growth. Then, the sugar starvation in fruits might stimulate the induction of plant hormone signal transduction (e.g., auxin and GA) and sucrose metabolism (e.g., *glycosyl hydrolase family 1*, *3*, *9*, *14*, *28*), and further enhance the activities of antioxidant enzymes (e.g., *POD44*) and TFs (e.g., *MYB39*, *AP2* and *bZIP43*). This process is also accompanied by other metabolisms and pathways, such as protein processing in endoplasmic reticulum and fatty acid metabolism as well as cell wall modification, and finally leads to the fruit abscission.

To conclude, our study provides the preliminary insights into the regulatory genes that possibly function in the second and third fruit drop stages of almond fruit development. Those genes were mainly involved in the carbon signaling, hormone and ROS pathways. The identification of these candidate genes could be helpful for understanding the molecular mechanisms involved in almond abscission. Therefore, further physiological and functional studies are required to clarify these findings in the future. Meanwhile, considering the correlation between gene patterns and observed morphological changes at 12 and 17 DAF, we suggest that more attention should be paid to these stages in cultivation. Furthermore, it will be informative to integrate our transcriptome data with trait-associated markers identified by previous population studies.

## Materials and methods

### Plant material and experimental design

The almond cultivar ‘ZhiPi’ was used for our study. The trees were planted in Shache county of Kashi city, Xinjiang province in 2001. They were grown in sandy soil at pH 7.9. The soil contained 11.8% organic matter, alkali nitrogen of 74.9 mg/kg, organophosphorus of 15.6 mg/kg, and quick-acting potassium of 144.2 mg/kg. The spacing of the trees was 6 × 7 m. All the almond trees were managed regularly.

From April to May in 2015, 30 almond trees with a consistent growth state were randomly selected for phenotypic investigation and sampling. The number of normal growing fruits and abnormal fruits at six time points (12, 17, 22, 27, 32 and 37 day after flowering, DAF) was counted, respectively. Meanwhile, the length of new shoot was measured for five replicates of each tree. The growth state of the fruits at 32 DAF in Fig. 1B was photographed in the sampling field. The sampling was conducted at 10 o'clock in the morning, and fruits without shade located in the southern part of the tree were selected. Normally developed and diapause atrophy fruits (abnormally developed) in the same branch were observed, recorded and sampled, respectively. For each replicate, 10–15 fruits were collected from each plant of the same cultivar. The fruits were then cut into small pieces and mixed, and sent for subsequent RNA extraction and further high-throughput sequencing.

### Total RNA extraction, cDNA library construction and RNA-seq

Total RNA was extracted from each fruit sample using RNAiso Plus (Takara). cDNA was generated in 25-μl reaction mixtures containing 2 μg DNAse I-treated RNA, 200 U M-MLV reverse transcriptase (Takara), 40 U Recombinant RNase Inhibitor (Takara) and 0.1 μΜ oligo (dT) 18 primer. Library construction and quality detection were completed by the Biomarker company (Beijing, China). RNA quality and integrity were analyzed using an Agilent Bioanalyzer 2100 system (Agilent Technologies, CA, USA). By using the TruSeq PE Cluster Kit v3-cBot-HS (Illumia), the index-coded samples were clustered following the manufacturer’s instructions. Finally, the libraries were loaded into an Hiseq 2500 (Illumina, USA) for pair-end model sequencing (101 bp).

### Bioinformatics analysis

We removed the RNA-seq reads that containing adaptors, with 5% sequencing errors and/or *q*-value less than 25, to obtain clean reads. Next, we performed de novo assembly using Trinity program^29^. The longest sequences of the redundant transcripts were considered as representative unigenes and subjected to downstream functional annotation and coding sequence (CDS) prediction.

The CDS were predicted using Trinotate v3.1.1 (https://trinotate.github.io/) method as follows: the unigenes were aligned to NCBI GenBank Non-Redundant Protein Sequences (NR) and Swiss-Prot databases, Clusters of Orthologous Groups (COG), eukaryotic Orthologous Groups (KOG), Evolutionary genealogy of genes: Non-supervised Orthologous Groups (eggNOG v4.5.1), Pfam database v31.0 (https://pfam.xfam.org), Gene Ontology (GO) and Kyoto Encyclopedia of Genes and Genomes (KEGG) by BLAST with *E*-value cut-off of 1 × 10^–5^. The best matches were used to predict the putative coding sequence region and determine the 5–3′orientation.

Clean reads were aligned back to the assembled unigenes using Bowtie2. The read counts estimated by RSEM software^61^ were normalized by DESeq2, and were used to calculate Fragments Per Kilobase of transcript per Million fragments mapped (FPKM) according to the effective length of genes and the library size (total number of reads). Then we estimated the significance of differential expression with DESeq2. Differentially expressed genes were selected with following criteria: *P*-value < 0.05 and |log2FoldChange|> 1 between two samples. GO enrichment analysis was subsequently performed for the DEGs using agriGO based on hyper geometric test (*P*-value < 0.05)^62^. KEGG enrichment analysis of the DEGs was carried out using KEGG Orthology^63^ and KOBAS 3.0 (Available online: https://kobas.cbi.pku.edu.cn) based on the hyper-geometric test (*P*-value < 0.05).The pipeline of the RNA-seq analysis could be found in Figure S7.

### Reverse transcription and real-time PCR

In this study, to reduce the variations caused by field environments, the RNA-seq samples were retrieved from three independent trees, and we used three biological replicates for the RT-qPCR assay. Two micrograms of total RNA for each sample were subjected to DNA digestion, and the mRNA were reverse-transcribed into cDNA with EasyScript One-Step gDNA Removal and cDNA Synthesis SuperMix Kit (Transgene Biotech, Beijing, China). The synthesized cDNA was diluted tenfold and 2 μL of the dilution was used for real-time PCR. The actin gene was set as the internal control, and the relative gene expression was quantified using the 2^−ΔΔCt^ method^64^. All RT-qPCR reactions were conducted in at least three biological replicates with an annealing temperature of 60 °C and a total of 40 cycles of amplification. The related primers used for real-time PCR are listed in Table S5.

### Statistical analysis

For the statistical analysis, R programming language (version 3.5.1) was used. Expression correlation was calculated with least squares linear regression in R. To test significance between the expression levels of normal and abnormal pairs, Student's *t-test* was performed.

## Supplementary information

Supplementary Information

Supplemental Table 1

Supplemental Table 2

Supplemental Table 3

Supplemental Table 4

Supplemental Table 5

## Data Availability

The RNA-seq datasets and the fastq-formatted sequences are available in in NCBI GEO database (https://www.ncbi.nlm.nih.gov/geo/) under accession GSE138034.

## References

[1] Gharaghani A, Solhjoo S, Oraguzie N (2017). A review of genetic resources of almonds and stone fruits (*Prunus* spp.) in Iran. Genet. Resour. Crop Evol..

[2] Company RSIG, Thomas M (2017). Almonds: Botany, Production and Uses.

[3] Gustafson W, Morrissey T, Bish C (1989). Plant exploration and germplasm collection of cold hardy woody plants for Nebraska from the People’s Republic of China.

[4] 4FAOSTAT. <https://www.fao.org/faostat/en/#data/QC> (2017).

[5] Godini A (2002). Almond fruitfulness and role of self-fertility. Acta Hort..

[6] Li C, Wang Y, Ying P, Ma W, Li J (2015). Genome-wide digital transcript analysis of putative fruitlet abscission related genes regulated by ethephon in litchi. Front. Plant Sci..

[7] Wong D, Gutierrez RL, Dimopoulos N, Gambetta G, Castellarin S (2016). Combined physiological, transcriptome, and cis-regulatory element analyses indicate that key aspects of ripening, metabolism, and transcriptional program in grapes (*Vitis vinifera* L.) are differentially modulated accordingly to fruit size. BMC Genom..

[8] Yuan R, Kender WJ, Burns JK (2003). Young fruit and auxin transport inhibitors affect the response of MatureValencia'Oranges to abscission materials via changing endogenous plant hormones. J. Am. Soc. Hortic. Sci..

[9] Lakso A, Wünsche J, Palmer J, Corelli Grappadelli L (1999). Measurement and modeling of carbon balance of the apple tree. HortScience.

[10] Quinlan JD, Preston AP (1971). The influence of shoot competition on fruit retention and cropping of apple trees. J. Horticult. Sci. Biotechnol..

[11] Zhu H (2011). Transcriptomics of shading-induced and NAA-induced abscission in apple (*Malus domestica*) reveals a shared pathway involving reduced photosynthesis, alterations in carbohydrate transport and signaling and hormone crosstalk. BMC Plant Biol..

[12] Li C (2013). De novo assembly and characterization of fruit transcriptome in Litchi chinensis Sonn and analysis of differentially regulated genes in fruit in response to shading. BMC Genom..

[13] Gómez-Cadenas A, Mehouachi J, Tadeo FR, Primo-Millo E, Talon M (2000). Hormonal regulation of fruitlet abscission induced by carbohydrate shortage in citrus. Planta.

[14] Yang Z (2015). Burst of reactive oxygen species in pedicel-mediated fruit abscission after carbohydrate supply was cut off in longan (*Dimocarpus longan*). Front. Plant Sci..

[15] Basak A (2011). Efficiency of fruitlet thinning in apple ‘Gala Must’by use of metamitron and artificial shading. J. Fruit Ornam. Plant Res.

[16] Byers R, Carbaugh D, Presley C, Wolf T (1991). The influence of low light on apple fruit abscission. J. Horticult. Sci..

[17] McArtney S, White M, Latter I, Campbell J (2004). Individual and combined effects of shading and thinning chemicals on abscission and dry-matter accumulation of ‘Royal Gala’apple fruit. J. Hortic. Sci. Biotechnol..

[18] Nicolás E, Lescourret F, Génard M, Bussi C, Besset J (2006). Does dry matter partitioning to fruit in early-and late-ripening peach (*Prunus persica*) cultivars confirm the branch autonomy theory?. J. Hortic. Sci. Biotechnol..

[19] Nzima MD, Martin GC, Nishijima C (1999). Effect of fall defoliation and spring shading on shoot carbohydrate and growth parameters among individual branches of alternate bearing Kerman'pistachio trees. J. Am. Soc. Hortic. Sci..

[20] Li C (2015). An improved fruit transcriptome and the identification of the candidate genes involved in fruit abscission induced by carbohydrate stress in litchi. Front. Plant Sci..

[21] Pertea M, Kim D, Pertea GM, Leek JT, Salzberg SL (2016). Transcript-level expression analysis of RNA-seq experiments with HISAT StringTie and Ballgown. Nat. Protoc..

[22] 22Henschel, R. *et al.* Trinity RNA-Seq assembler performance optimization. *XSEDE’12 Proceedings of the 1st Conference of the Extreme Science and Engineering Discovery Environment: Bridging from the eXtreme to the campus and beyond (Chicago, Illinois, USA, July 16–20, 2012)* (2012).

[23] Karimi M (2016). The small-RNA profiles of almond (Prunus dulcis Mill.) reproductive tissues in response to cold stress. PLoS ONE.

[24] Wang J (2015). Mongolian almond (Prunus mongolica Maxim): the morpho-physiological, biochemical and transcriptomic response to drought stress. PLoS ONE.

[25] Mousavi S (2014). De novo transcriptome assembly and comparative analysis of differentially expressed genes in Prunus dulcis Mill. in response to freezing stress. PLoS ONE.

[26] Gómez EM, Buti M, Sargent DJ, Dicenta F, Ortega E (2019). Transcriptomic analysis of pollen-pistil interactions in almond (Prunus dulcis) identifies candidate genes for components of gametophytic self-incompatibility. Tree Genet. Genomes.

[27] Sánchez-Pérez R (2019). Mutation of a bHLH transcription factor allowed almond domestication. Science.

[28] Prudencio, A., Dicenta, F. & Martínez-Gómez, P. Gene expression analysis of flower bud dormancy breaking in almond using RNA-Seq. *In Proceedings of the VII International Symposium on Almonds & Pistachios (Adelaida, Australia, November 5–9, 2017)*, 119–124 (2017).

[29] Haas BJ (2013). De novo transcript sequence reconstruction from RNA-seq using the Trinity platform for reference generation and analysis. Nat. Protoc..

[30] Velasco D, Hough J, Aradhya MK, Rossibarra J (2016). Evolutionary genomics of peach and almond domestication. G3 Genes Genomes Genet..

[31] Yu Y (2018). Genome re-sequencing reveals the evolutionary history of peach fruit edibility. Nat. Commun..

[32] Verde I (2017). The Peach v2.0 release: High-resolution linkage mapping and deep resequencing improve chromosome-scale assembly and contiguity. BMC Genom..

[33] Trapnell C (2010). Transcript assembly and quantification by RNA-Seq reveals unannotated transcripts and isoform switching during cell differentiation. Nat. Biotechnol..

[34] Love MI, Huber W, Anders S (2014). Moderated estimation of fold change and dispersion for RNA-seq data with DESeq2. Genome Biol..

[35] De Jong M (2015). Solanum lycopersicum AUXIN RESPONSE FACTOR 9 regulates cell division activity during early tomato fruit development. J. Exp. Bot..

[36] Groszmann M, Paicu T, Smyth DR (2008). Functional domains of SPATULA, a bHLH transcription factor involved in carpel and fruit development in Arabidopsis. Plant J..

[37] Seymour GB, Østergaard L, Chapman NH, Knapp S, Martin C (2013). Fruit development and ripening. Annu. Rev. Plant Biol..

[38] Zheng Q, Wang X-J (2008). GOEAST: A web-based software toolkit for gene ontology enrichment analysis. Nucleic Acids Res.

[39] Goswami S (2016). SSH analysis of endosperm transcripts and characterization of heat stress regulated expressed sequence tags in bread wheat. Front. Plant Sci..

[40] Wickham H (2016). ggplot2: elegant graphics for data analysis.

[41] Pollard M, Beisson F, Li Y, Ohlrogge JB (2008). Building lipid barriers: Biosynthesis of cutin and suberin. Trends Plant Sci..

[42] Gloster TM (2014). Advances in understanding glycosyltransferases from a structural perspective. Curr. Opin. Struct. Biol..

[43] Zhong R, Ye Z-H (2003). Unraveling the functions of glycosyltransferase family 47 in plants. Trends Plant Sci..

[44] Del Pozo JC (1999). A type 5 acid phosphatase gene from Arabidopsis thaliana is induced by phosphate starvation and by some other types of phosphate mobilising/oxidative stress conditions. Plant J..

[45] Zhang W, Gruszewski HA, Chevone BI, Nessler CL (2008). An Arabidopsis purple acid phosphatase with phytase activity increases foliar ascorbate. Plant Physiol..

[46] Jian-Jie G (2011). Forced expression of Mdmyb10, a myb transcription factor gene from apple, enhances tolerance to osmotic stress in transgenic Arabidopsis. Mol. Biol. Rep..

[47] Wang X, Chen X, Yang T, Cheng Q, Cheng Z (2017). Genome-wide identification of bZIP family genes involved in drought and heat stresses in strawberry (Fragaria vesca). Comp. Funct. Genom..

[48] Zhou Y (2014). Transcriptome analysis and transient transformation suggest an ancient duplicated MYB transcription factor as a candidate gene for leaf red coloration in peach. BMC Plant Biol..

[49] Rai MK, Shekhawat NS (2014). Recent advances in genetic engineering for improvement of fruit crops. Plant Cell Tissue Organ Cult..

[50] Zhang Y (2015). Transcript quantification by RNA-seq reveals differentially expressed genes in the red and yellow fruits of Fragaria vesca. PLoS ONE.

[51] Fang Z-Z, Zhou D-R, Ye X-F, Jiang C-C, Pan S-L (2016). Identification of candidate anthocyanin-related genes by transcriptomic analysis of ‘Furongli’plum (Prunus salicina Lindl.) during fruit ripening using RNA-seq. Front. Plant Sci..

[52] Rolland F, Baena-Gonzalez E, Sheen J (2006). Sugar sensing and signaling in plants: conserved and novel mechanisms. Annu. Rev. Plant Biol..

[53] Morandi B (2011). Shading decreases the growth rate of young apple fruit by reducing their phloem import. Sci. Hortic..

[54] Tombesi S, Lampinen B, Metcalf S, DeJong T (2016). Yield in almond is related more to the abundance of flowers than the relative number of flowers that set fruit. Calif. Agric..

[55] 55Polito, V., Micke, W. & Kester, D. Bud development, pollination and fertilization. *Almond Production Manual. University of California Publication*, 98–102 (1996).

[56] Sakai WS, Nagao MA (1985). Fruit growth and abscission in Macadamia imtegrifolia. Physiol. Plant..

[57] McAtee P, Karim S, Schaffer RJ, David K (2013). A dynamic interplay between phytohormones is required for fruit development, maturation, and ripening. Front. Plant Sci..

[58] Srivastava A, Handa AK (2005). Hormonal regulation of tomato fruit development: A molecular perspective. J. Plant Growth Regul..

[59] Estornell LH, Agusti J, Merelo P, Talon M, Tadeo FR (2013). Elucidating mechanisms underlying organ abscission. Plant Sci..

[60] Koukourikou-Petridou MA (2003). The relation between the levels of extractable and diffusible IAA in almond fruits and their “June drop”. Plant Growth Regul..

[61] Li B, Dewey CN (2011). RSEM: Accurate transcript quantification from RNA-Seq data with or without a reference genome. BMC Bioinform..

[62] Tian T (2017). AgriGO v20: a GO analysis toolkit for the agricultural community, 2017 update. Nucleic Acids Res.

[63] Kanehisa M, Goto S, Kawashima S, Okuno Y, Hattori M (2004). The KEGG resource for deciphering the genome. Nucleic Acids Res.

[64] Livak KJ, Schmittgen TD (2001). Analysis of relative gene expression data using real-time quantitative PCR and the 2−ΔΔCT method. Methods.

