# Supplementary material for: Developmental transcriptome profiling uncovered carbon signaling genes associated with almond fruit drop
Source: Sci Rep. 2021 Feb 9;11:3401. doi: 10.1038/s41598-020-69395-z (PMC7873282; doi:10.1038/s41598-020-69395-z)

Developmental Transcriptome Profiling Uncovered Carbon Signaling Genes Associated with Almond fruit drop

Chunmiao Guo[1,2†], Yu Wei [3†], Bo Yang[2†], Mubarek Ayup[2], Ning Li[2], Jun Liu[3], Huan Wang[4*] and Kang Liao[1*]

[1] College of Forestry and Horticulture, Xinjiang Agricultural University, Urumqi 830052, China; chunmiaoguo@126.com (C.G.); 13899825018@163.com (K.L)

[2] Institute of Horticultural Crops, Xinjiang Academy of Agricultural Sciences, Urumqi 830091, China; liningbio@163.com (N.L.); yangboyys@163.com (B.Y.); mubarek@ms.xjb.ac.cn (M. A.)

[3] National Key Facility for Crop Resources and Genetic Improvement, Institute of Crop Science, Chinese Academy of Agricultural Sciences, Beijing 100081, China; yu.wei2005@outlook.com (Y.W.); liujun@caas.cn (J.L.)

[4] Biotechnology Research Institute, Chinese Academy of Agricultural Sciences, Beijing 100081, China; wanghuan@caas.cn (H.W.)

***** Correspondence: wanghuan@caas.cn (H.W.); 13899825018@163.com (K.L)

† These authors have contributed equally to this work.

Supplementary information

Figure S1: The characteristics of almond fruits at six stages from the 7 to 32 DAF.

Figure S2: The morphological evaluation of almond fruits from 12 to 42 DAF. (A) The fruit weight of normal and abnormal fruits. (B) The longitudinal diameter of abnormal fruit. (C) The Transversal diameter of abnormal fruit.

Figure S3: Venn diagram showed the DEGs identified in different groups.

Figure S4A: Similar expression profiles of DEGs between normal and abnormal fruits; S4B: Other six different expression patterns of DEGs between normal and abnormal fruits.

Figure S5: KEGG pathway of "carbon metabolism".

Figure S6: Validation of gene expression by RT-qPCR. (A) Overall expression levels of eight DEGs across different developmental stages. Bars give standard errors (n=3). N, normal; A, abnormal. Blue, RT-qPCR analysis; red, RNA-seq analysis; P-values indicate statistical significance (*P < 0.05, **P < 0.01, ***P < 0.001). (B) Comparison of RNA-seq and RT-qPCR data. A correlation coefficient of R = 0.754 (P-value <0.001) was observed between the RNA-seq and RT-qPCR data of all the seven DEGs across different developmental stages.

Figure S7: The pipeline of the RNA-seq analysis.

Figure S1

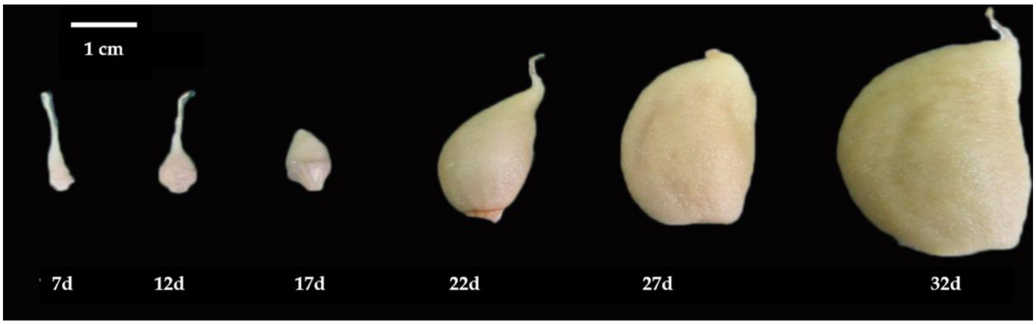

Figure S2

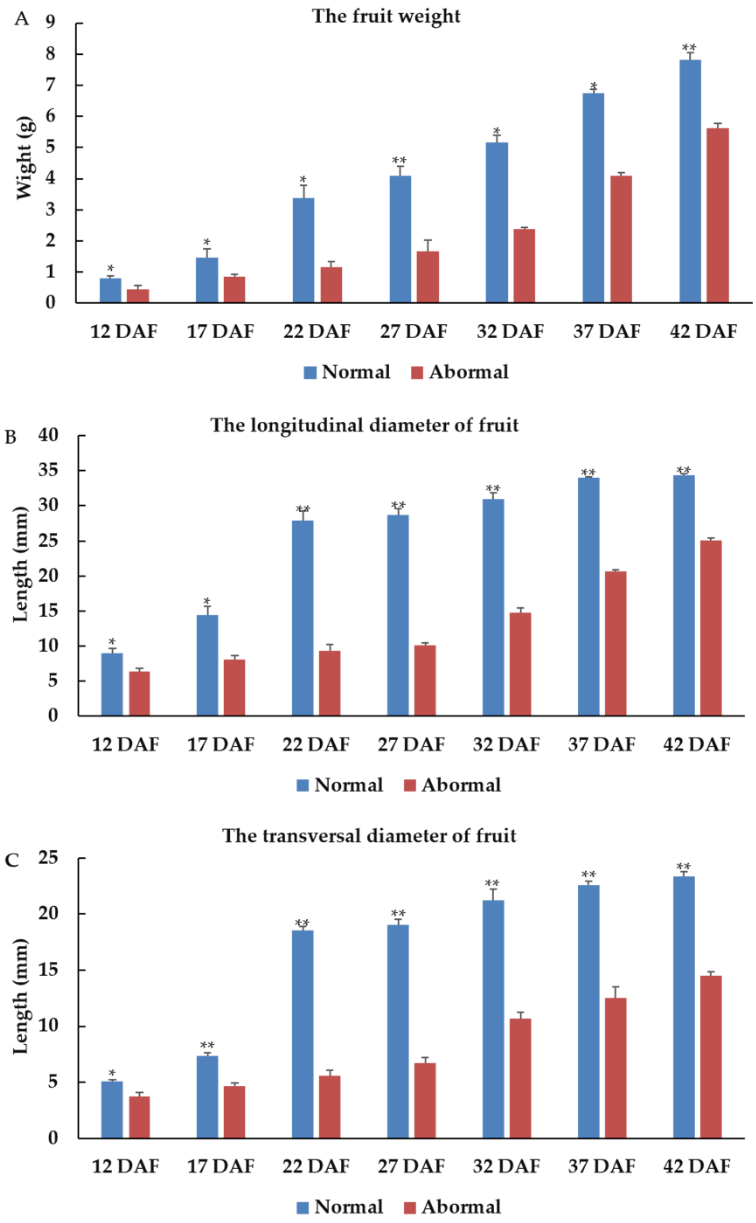

Figure S3

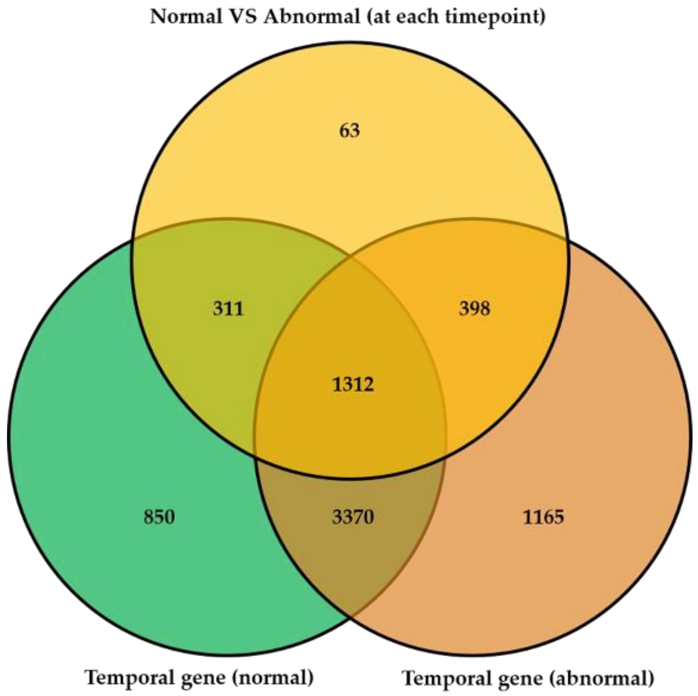

Normal VS Abnormal (at each timepoint)
63
311
398
1312
850
3370
1165
Temporal gene (normal)
Temporal gene (abnormal)

Figure S4

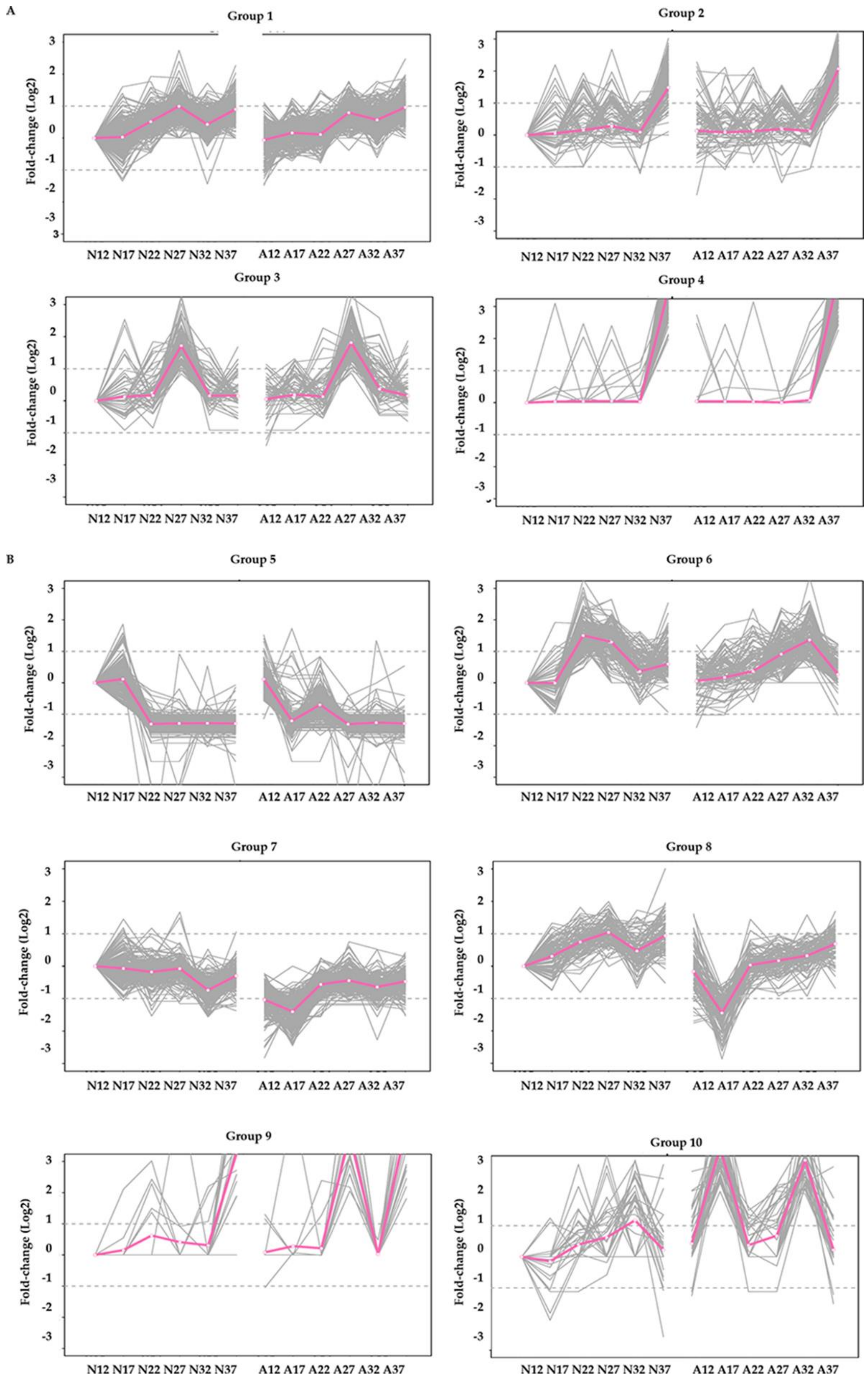
A
B
Group 1
Group 2
Group 3
Group 4
Group 5
Group 6
Group 7
Group 8
Group 9
Group 10
Fold-change (Log2)
N12 N17 N22 N27 N32 N37
A12 A17 A22 A27 A32 A37

Figure S5

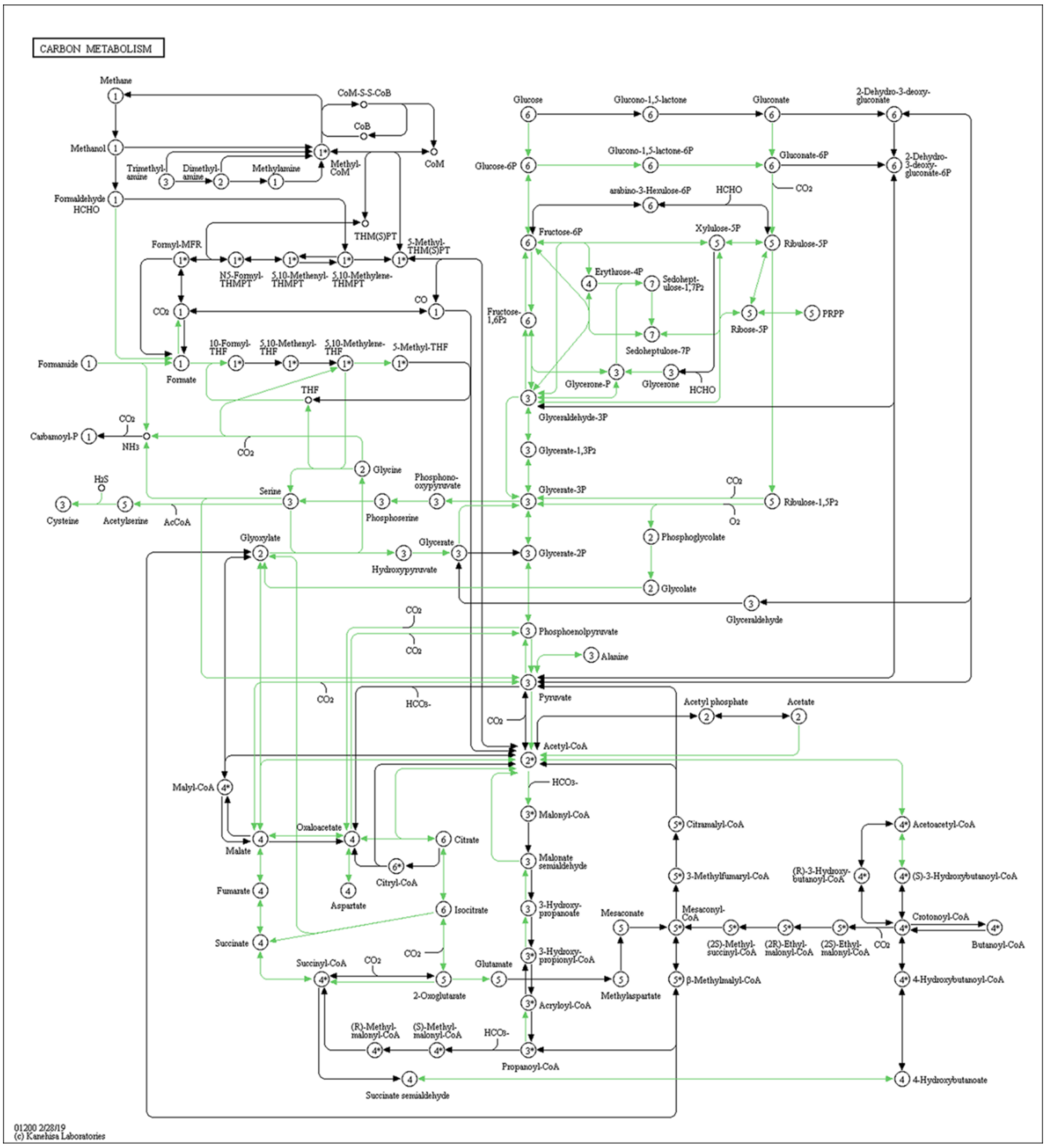
CARBON METABOLISM
Methane
CoM-S-S-CoB
CoB
Methanol
CoM
Trimethyl-amine
Dimethyl-amine
Methylamine
Methyl-CoM
Formaldehyde HCHO
THM(S)PT
Formyl-MFR
5-Methyl-THM(S)PT
N5-Formyl-THMPT
5,10-Methenyl-THMPT
5,10-Methylene-THMPT
CO_2
CO
10-Formyl-THF
5,10-Methenyl-THF
5,10-Methylene-THF
5-Methyl-THF
Formamide
Formate
THF
Carbamoyl-P
NH3
H_2S
Cysteine
Acetylserine
AcCoA
Serine
Glycine
Phosphono-oxypyruvate
Phosphoserine
Glyoxylate
Glycerate
Hydroxypyruvate
Glucose
Glucono-1,5-lactone
Gluconate
2-Dehydro-3-deoxy-gluconate
Glucose-6P
Glucono-1,5-lactone-6P
Gluconate-6P
2-Dehydro-3-deoxy-gluconate-6P
arabino-3-Hexulose-6P
HCHO
Fructose-6P
Xylulose-5P
Ribulose-5P
Erythrose-4P
Sedohept-ulose-1,7P2
Fructose-1,6P2
Ribose-5P
PRPP
Sedoheptulose-7P
Glycerone-P
Glycerone
Glyceraldehyde-3P
Glycerate-1,3P2
Glycerate-3P
Ribulose-1,5P2
O_2
Phosphoglycolate
Glycerate-2P
Glycolate
Glyceraldehyde
Phosphoenolpyruvate
Alanine
Pyruvate
HCO_3^-
Acetyl phosphate
Acetate
Acetyl-CoA
Malyl-CoA
Malonyl-CoA
Oxaloacetate
Citrate
Malate
Citryl-CoA
Malonate semialdehyde
Citramalyl-CoA
Acetoacetyl-CoA
Fumarate
Aspartate
Isocitrate
3-Hydroxy-propanoate
3-Methylfumaryl-CoA
(R)-3-Hydroxy-butanoyl-CoA
(S)-3-Hydroxybutanoyl-CoA
Mesaconate
Mesaconyl-CoA
Crotonoyl-CoA
Succinate
3-Hydroxy-propionyl-CoA
(2S)-Methyl-succinyl-CoA
(2R)-Ethyl-malonyl-CoA
(2S)-Ethyl-malonyl-CoA
Butanoyl-CoA
Succinyl-CoA
Glutamate
2-Oxoglutarate
Methylaspartate
β-Methylmalyl-CoA
4-Hydroxybutanoyl-CoA
Acryloyl-CoA
(R)-Methyl-malonyl-CoA
(S)-Methyl-malonyl-CoA
Propanoyl-CoA
Succinate semialdehyde
4-Hydroxybutanoate
01200 2/28/19
(c) Kanehisa Laboratories

Figure S6A

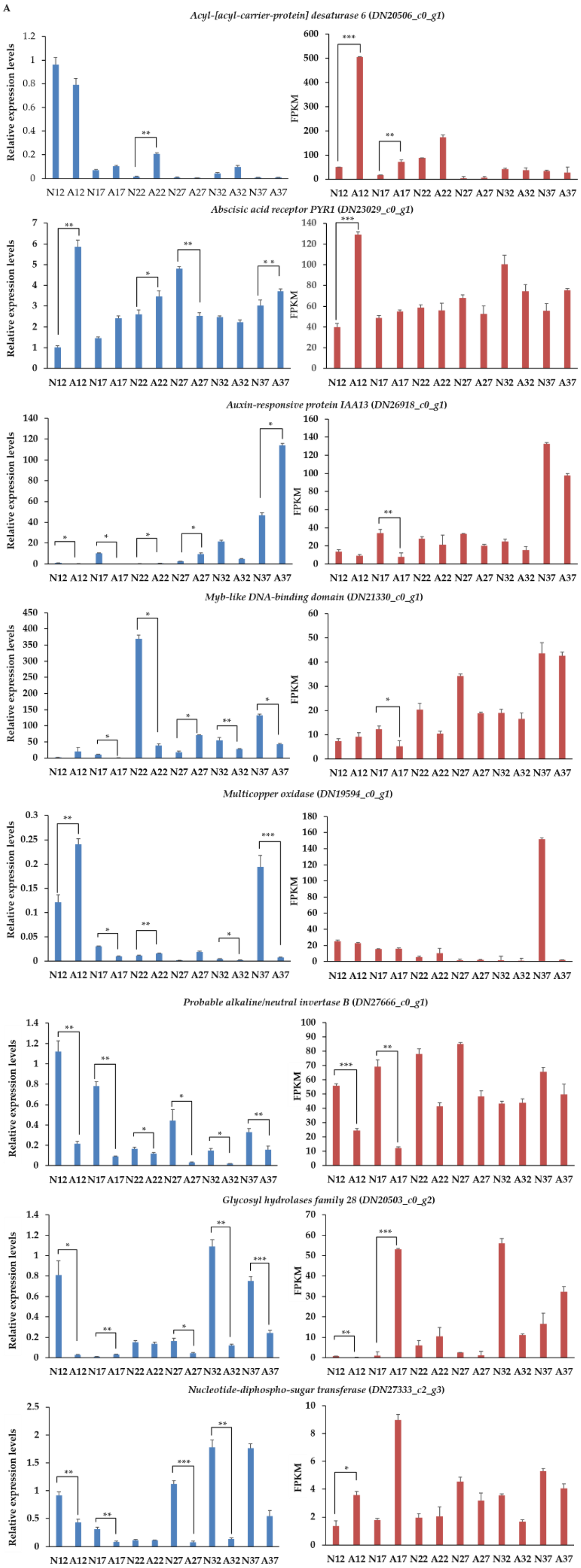
A
Acyl-[acyl-carrier-protein] desaturase 6 (DN20506_c0_g1)
Abscisic acid receptor PYR1 (DN23029_c0_g1)
Auxin-responsive protein IAA13 (DN26918_c0_g1)
Myb-like DNA-binding domain (DN21330_c0_g1)
Multicopper oxidase (DN19594_c0_g1)
Probable alkaline/neutral invertase B (DN27666_c0_g1)
Glycosyl hydrolases family 28 (DN20503_c0_g2)
Nucleotide-diphospho-sugar transferase (DN27333_c2_g3)
Relative expression levels
FPKM
N12 A12 N17 A17 N22 A22 N27 A27 N32 A32 N37 A37

Figure 6B

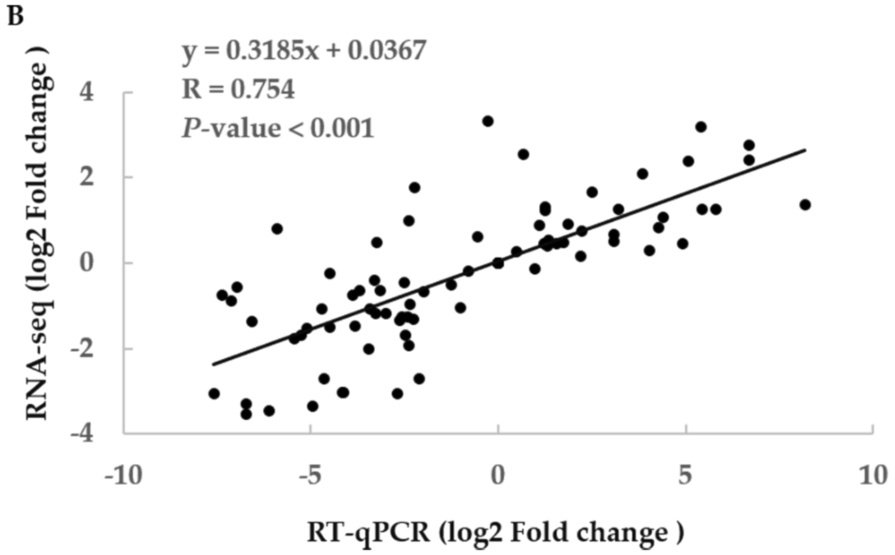

Figure S7

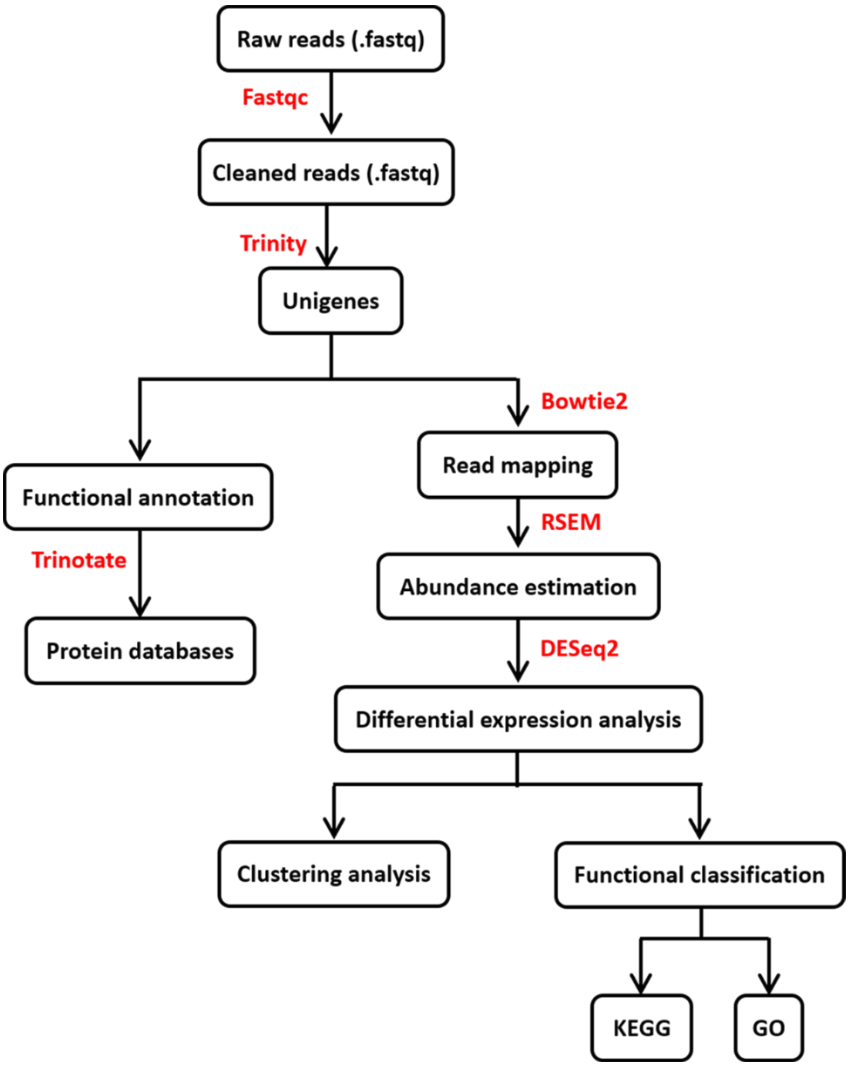

Supplement: Supplementary file 1 — Supplementary Information [file 41598_2020_69395_MOESM1_ESM.pdf]
